# Transcriptome analysis of *Corynebacterium glutamicum* in the process of recombinant protein expression in bioreactors

**DOI:** 10.1371/journal.pone.0174824

**Published:** 2017-04-03

**Authors:** Yang Sun, Wenwen Guo, Fen Wang, Chunjun Zhan, Yankun Yang, Xiuxia Liu, Zhonghu Bai

**Affiliations:** 1 National Engineering Laboratory for Cereal Fermentation Technology, Jiangnan University, Wuxi, China; 2 The Key Laboratory of Industrial Biotechnology, Ministry of Education, School of Biotechnology, Jiangnan University, Wuxi, China; 3 The Key Laboratory of Carbohydrate Chemistry and Biotechnology, Ministry of Education, School of Biotechnology, Jiangnan University, Wuxi, China; National Renewable Energy Laboratory, UNITED STATES

## Abstract

*Corynebacterium glutamicum* (*C*. *glutamicum*) is a favorable host cell for the production of recombinant proteins, such as important enzymes and pharmaceutical proteins, due to its excellent potential advantages. Herein, we sought to systematically explore the influence of recombinant protein expression on the transcription and metabolism of *C*. *glutamicum*. Two *C*. *glutamicum* strains, the wild-type strain and an engineered strain expressing enhanced green fluorescent protein (EGFP), were cultured in parallel in 5-L bioreactors to study the change in metabolism in the process of EGFP expression. The results revealed that EGFP expression had great effects on the growth and metabolism of *C*. *glutamicum* and contributed to metabolism-like anaerobic conditions as follows: glycolysis was enhanced, the TCA cycle was shunted, and Glu, Val, Met, lactate and acetate were accumulated to produce sufficient ATP for EGFP production and transfer. Many differentially expressed genes related to ribosomal protein, transcriptional regulators, and energy metabolism were found to be expressed in the presence of EGFP, laying the foundation for identifying genomic loci to change the flow of the host cell metabolism to improve the ability of expressing foreign proteins in *C*. *glutamicum*.

## Introduction

Since 1957, when the Gram-positive bacterium *Corynebacterium glutamicum* (*C*. *glutamicum*) was isolated due to its ability to excrete large amounts of L-glutamate [1], *C*. *glutamicum* has been thoroughly investigated and used as a generally regarded-as-safe organism in the fermentation industry. Within the last decades, *C*. *glutamicum* was proven to be an excellent production platform for amino acids and organic acids, and a promising alternative bacterial host for the expression of recombinant proteins due to its unique characteristics. First, *C*. *glutamicum* is non-pathogenic and does not produce endotoxins. Furthermore *C*. *glutamicum* could secrete properly folded proteins into the culture medium and lacks detectable extracellular hydrolysis activity, activities that increase the subsequent purification efficiency [2]. With these advantages, *C*. *glutamicum* has been widely used to express recombinant proteins such as staphylococcal nuclease [3], transglutaminase [4], human epidermal growth factor [5], antibody fragment [6], green fluorescent protein (GFP) [7] and α-amylase [8], et al.

When *C*. *glutamicum* expresses recombinant proteins, it has several intrinsic disadvantages, such as a low transformation efficiency, few available expression vectors [6], and a lower yield than that of *Escherichia coli*. Tremendous efforts have been put forth into the genetic modification and cultivation of *C*. *glutamicum* to avoid these issues—for example, the development of sophisticated tools for genetic manipulation, the engineering of natural and endogenous promoters and signal peptides, the optimization of codon usage, and the knockout of some proteases to reduce the degradation of recombinant proteins [9–13]. In addition, recombinant protein expression is also an ATP-consuming process, and an adequate supply of ATP could appropriately improve the expression level of recombinant proteins [14]. Due to the complex process of heterologous protein expression and energy metabolism of *C*. *glutamicum*, it is not easy to improve the production of heterologous protein simply through increasing the ATP level but through identifying the positively correlated genes between them.

In this study, to systematically explore the effect of enhanced green fluorescent protein (EGFP) expression on the genetic regulation and metabolism of *C*. *glutamicum*, we compared *C*. *glutamicum* Egfp and *C*. *glutamicum* BZH 001 in a 5-L bioreactor through RNA-seq. Subsequently, the mechanistic correlation among these changes and EGFP expression was studied to understand the metabolism mechanism in the process of heterologous protein expression and to identify key nodes to reform the genomic loci to improve the ability to express foreign proteins. In general, the study of metabolism and transcriptome regulation in the heterologous protein expression of *C*. *glutamicum* has high academic value and broad application prospects.

## Materials and methods

### Strains and cultivation

The wild-type strain used in this work was *C*. *glutamicum* CGMCC1.15647 (*C*. *glutamicum* BZH 001) donated by Zhangjiagang Huachang Pharmaceutical Co., Ltd. (Zhangjiagang, China). Its derivatives *C*. *glutamicum* pXMJ19-EGFP (*C*. *glutamicum* EGFP) was constructed as the following. EGFP gene was amplified by PCR using F primer (5’-CCAAGCTTAAAGGAGGACAACTAATGGTGAGCAAGGGCG-3’) and R primer (5’-CCCGGATCCTTACTTGTACAGCTCGTCCATG-3’) with plasmid PEGFP-N1 as the template. The amplified DNA fragment was introduced into the PXMJ19 by *Hind* III and *Bam*H I digestion and ligation. The lacIq gene was removed from PXMJ19 to make it a constitutive expression vector. The resulting plasmid PXMJ19-EGFP was introduced into *C*. *glutamicum* BZH 001. The EGFP gene was constitutive express by *tac* promoter witha conserved SD sequence (aaaggagga). The physical map of PXMJ19-EGFP was shown in S1 Fig [15].

The strain was grown on seed medium (25 g of glucose, 30 g of corn syrup, 20 g of (NH_4_)_2_SO_4_, 1 g of MgSO_4_, and 1 g of KH_2_PO_4_ per liter of distilled H_2_O; pH 6.8) in a shaker for 12 h at 30°C and 220 rpm. Next, the seed was transferred to the second seed medium in a baffled-bottom flask at 30°C, pH 6.8, for 48 h before transferring to fermentation medium in a 5-L fermenter (Applikon EZ-control, Netherlands). Fed batches were conducted in parallel with three biological replicates of *C*. *glutamicum* BZH 001, and *C*. *glutamicum* EGFP under 30% dissolved oxygen (DO). Glucose (300 g/L) was added into the fermenter at the rate of 10 mL/h 16 h after inoculation to avoid nutrient shortage. DO calibration and control: In this experiment, 100% DO was calibrated after equilibration for 5 h with an aeration rate of 3 L/min and an agitator speed of 400 rpm at 30°C. The DO level (percentage of air saturation) was controlled at constant value by varying the agitation from 400 to 1000 rpm and supplying pure oxygen through a solenoid valve. The DO value was allowed to fluctuate by less than 4%. The medium was composed of 30 g of glucose, 15 g of corn syrup, 20 g of (NH_4_)_2_SO_4_, 1 g of MgSO_4_, and 1 g of KH_2_PO_4_ per liter of distilled H_2_O at pH 6.8. The samples collected at 20 h from three independent batches were used for RNA sequencing and quantitative real-time RT-PCR analysis. Samples for metabolism analysis were collected every 4 h during the entire fermentation process. All samples were quenched in liquid nitrogen immediately after collection and were then stored at -80°C before analysis.

### Biomass and concentration of glucose

Biomass formation was followed by measuring the OD_600_ and by determining the cell dry weight. The concentration of glucose was measured using the 3, 5-dinitrosalicylic acid (DNS) method [16].

### HPLC analyses

The determination of amino acids and organic acids was performed as described previously [17, 18] by 1260 Agilent system (Agilent Technologies Inc., USA) and SHIMADZU HPLC system (SHIMADZU Corp., Japan).

### Quantification of enzyme activities

Quantification of the pyruvate carboxylase (PC), isocitrate dehydrogenase (ICDH), glutamate dehydrogenase (GDH) activities was performed according to modified protocols. The protein concentration was quantified using the Bradford assay. Specific activities were calculated as follows: a_s_ = a/TP [17].

### Quantification of energy

ATP was measured using an ATP Assay Kit (Beyotime, China).

### Transcriptome analysis by RNA-seq

The harvesting of total RNA and the RNA-Seq protocol were described previously [19]. The clean reads were exported in the FASTQ format and were deposited in the National Center for Biotechnology Information (NCBI) Sequence Read Archive (SRA) with the accession numbers GSE77502/GSE87077. The clean reads were aligned to the reference sequences (*C*. *glutamicum* ATCC13032 genome) by SOAPaligner/SOAP2 [20].

The alignment data were used to calculate the distribution of reads on reference genes and to perform coverage analysis. After the alignment results passed QC, downstream analysis was performed, including gene expression level analysis, differential expression analysis, pathway enrichment analysis, and gene ontology (GO) enrichment analysis. RPKM (reads per kb per million reads) was used for the gene expression level calculation [19, 21, 22].

### Quantitative real-time PCR

RNA was quantified using a NanoDrop ND-500 spectrophotometer and was used for cDNA synthesis. Real-time RT-PCR was performed using an ABI stepone Plus Real-Time PCR System and the SYBR RT-PCR Kit. The *16s RNA* gene was used as an internal control. Eight genes were chosen for expression analysis, and the primers are shown in S1 Table. The amplification conditions were as follows: 30 s at 95°C, 40 cycles of 5 s at 95°C, 30 s at 60°C, and 20 s at 72°C. The relative gene expression levels were calculated using the 2^-ΔΔCt^ method. Each experiment was repeated three times.

### Method of MVDA (PCA, OPLS-DA), Heatmap

MVDA was carried out by SIMCA-P 14(MKS UmetricsAB, Sweden) as the method described previously [19, 23].

Heatmap was performed by cluster software [24] and Java Treeview software [25]. The RPKM was deal by hierarchical clustering and Euclidean distance.

## Results

### Transcriptome sequencing (RNA-seq) output and expression annotation

In this study, three fermentation batches were conducted using *C*. *glutamicum* BZH 001 and *C*. *glutamicum* EGFP, respectively. Transcriptome analysis was performed on *C*. *glutamicum* BZH 001 and *C*. *glutamicum* EGFP to elucidate the relationship between heterologous protein expression and metabolism. Samples harvested at 20 h in fermenters were used for RNA-seq. At that point, the cells remained at the early stage of the stable phase. We studied the changes in gene expression from the samples taken after 20 h of fermentation from cultures under 30% DO concentrations. Each sample had three independent reproducible biological replicates; the average coefficient of variation (CV) was lower than 2.50% (S2 Table). After the QC step (analysis of base composition and quality), the raw reads were qualified and filtered into clean reads and then were mapped to the *C*. *glutamicum* ATCC13032 genome (The accession number is NC_003450.3). The total reads generated in each sample library ranged from 19,321,622 to 27,005,014. In total, approximately more than 16,300,000 reads were uniquely mapped to the genome, and their percentage in total reads was above 84% (S3 Table). Next, NOIseq was used to deal with clean data of the three duplicates to identify the differentially expressed genes (DEGs). An absolute value of the log2 ratio > 1 and FDR<0.05 were used to select genes with significant differential expression. When the *C*. *glutamicum* BZH 001 group was set as the control, 244 genes were significantly down-regulated, and 319 genes were up-regulated; 14 genes with |log2 ratio| > 4 were analyzed here. Four of the 14 DEGs (NCgl1413, NCgl2845, NCgl2837 and NCgl0910) were related to a hypothetical protein; thus, the focus was on the others. Among the 10 DEGs, NCgl0055 had a potential function in transcription regulation, and 5 genes had no gene annotation; thus, the other 4 DEGs (NCgl0487, NCgl0909, NCgl0833 and NCgl0303) were further analyzed. NCgl0487, encoding the 50S ribosomal protein L3 (rplC), was almost 95-fold down-regulated. rplC is a ribosomal protein involved in the translation elongation process, and its down-regulation indicates that fewer ribosomes are being produced to translate EGFP protein. NCgl0833 encoding the 50S ribosomal protein L33 (rpmG) was also down-regulated by 24.18-fold, and it was identified as a zinc-binding domain. NCgl0909 encoding the ABC transporter ATPase was significantly up-regulated. ABC transporters are members of a transport system superfamily, and ATPase is a subunit of ABC transporters that utilizes the energy of ATP binding and hydrolysis to transport various substrates across cellular membranes. The up-regulation of this gene in the *C*. *glutamicum* EGFP group indicated that the transmembrane transportation of some substrates was activated, possibly to transport EGFP. NCgl0303 encoding a cold shock protein was up-regulated. Shock protein, part of the CspA family, is the major cell surface protein, along with CspB. It was investigated that CspB mutation in *C*. *glutamicum* may increase the secretion of the antibody Fab fragment [13]; thus, up-regulated CspA may perform similarly in the *C*. *glutamicum* EGFP group.

### Analysis of Gene Ontology (GO) functional enrichment and pathway enrichment

**Fig 1:**
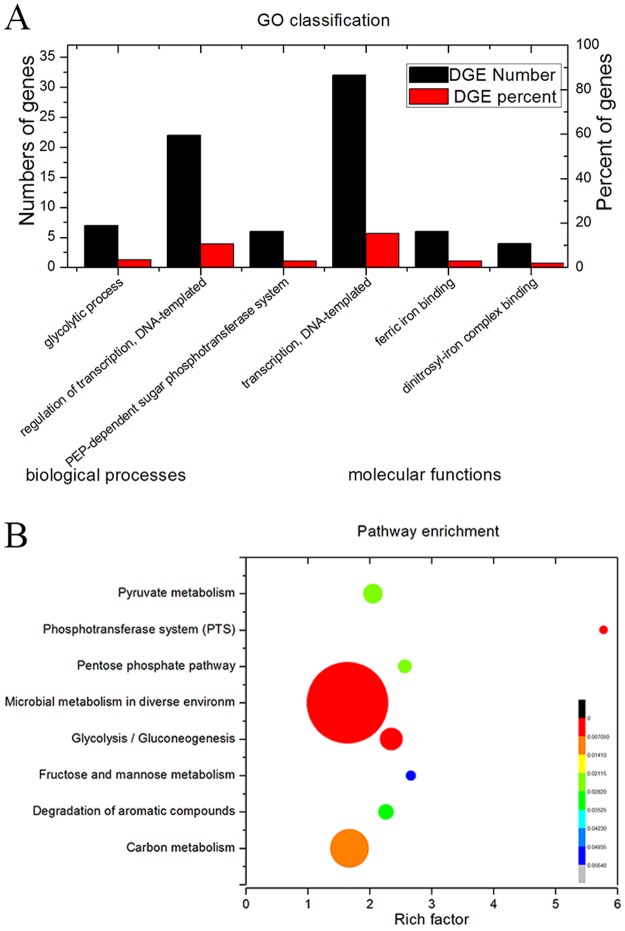
GO classification and pathway enrichment. A: GO classification of different expression genes. B: Pathways enrichment of different expression genes.

GO enrichment was performed to identify the potential function of the DEGs of the *C*. *glutamicum* EGFP group. The DEGs were annotated using GO annotations of GO term findings (https://david.ncifcrf.gov/gene2gene.jsp). Annotated genes were enriched with a corrected p-value ≤0.05. The DEGs could be categorized into two functional groups belonging to two main GO ontologies: biological processes (4) and molecular functions (2). The GO annotations belonging to the biological processes were GO:0009401-phosphoenolpyruvate-dependent sugar phosphotransferase system, GO:0006355-regulation of transcription, DNA-templated, GO:0006096-glycolytic process, and GO:0006351-transcription, DNA-templated. The molecular functions of GO ontologies were GO:0008199-ferric iron binding and GO:0035731-dinitrosyl-iron complex binding (Fig 1A). There were 32 genes clustered in transcription and 22 genes in the regulation of transcription. Each DEG was subjected to pathway analysis using the KEGG (Kyoto Encyclopedia of Genes and Genomes) database (http://www.kegg.jp/kegg/pathway.html) to explore the biological implications. In addition, we generated a scatter plot of the KEGG enrichment results (Fig 1B). A smaller rich factor indicates greater intensiveness, and a larger plot indicates more enrichment of genes. The p-value ranged from 0 to 0.056. Fig 1 shows the enrichment pathways of the *C*. *glutamicum* EGFP group. Specifically, the pathway terms enriched with the largest number of DEGs annotated were microbial metabolism in diverse environments; it was an important pathway of energy metabolism, substance metabolism and transcription and there were 46 genes annotated in this pathway. Heterologous protein expression needs many metabolic precursors and/or cofactors; thus, the pathway terms of Glycolysis/Gluconeogenesis, Carbon metabolism, Pentose phosphate pathway and Pyruvate metabolism were enriched. These pathways are important pathways to synthesize all required amino acid and ATP necessary to form substrates of the target protein. Five genes of the Phosphotransferase system (PTS) were enriched, and the p-value of this pathway was 0.0036; PTS could efficiently transport different sugars. Anda et al. replaced the glucose PTS with alternate glucose transport activity on growth kinetics and acetate accumulation to improve the production of recombinant proteins [26]. Thus, changing the activity of PTS is an important method to improve the cellular performance in recombinant protein production systems.

### Expression of the key genes of *C*. *glutamicum* BZH 001 and *C*. *glutamicum* EGFP

**Fig 2:**
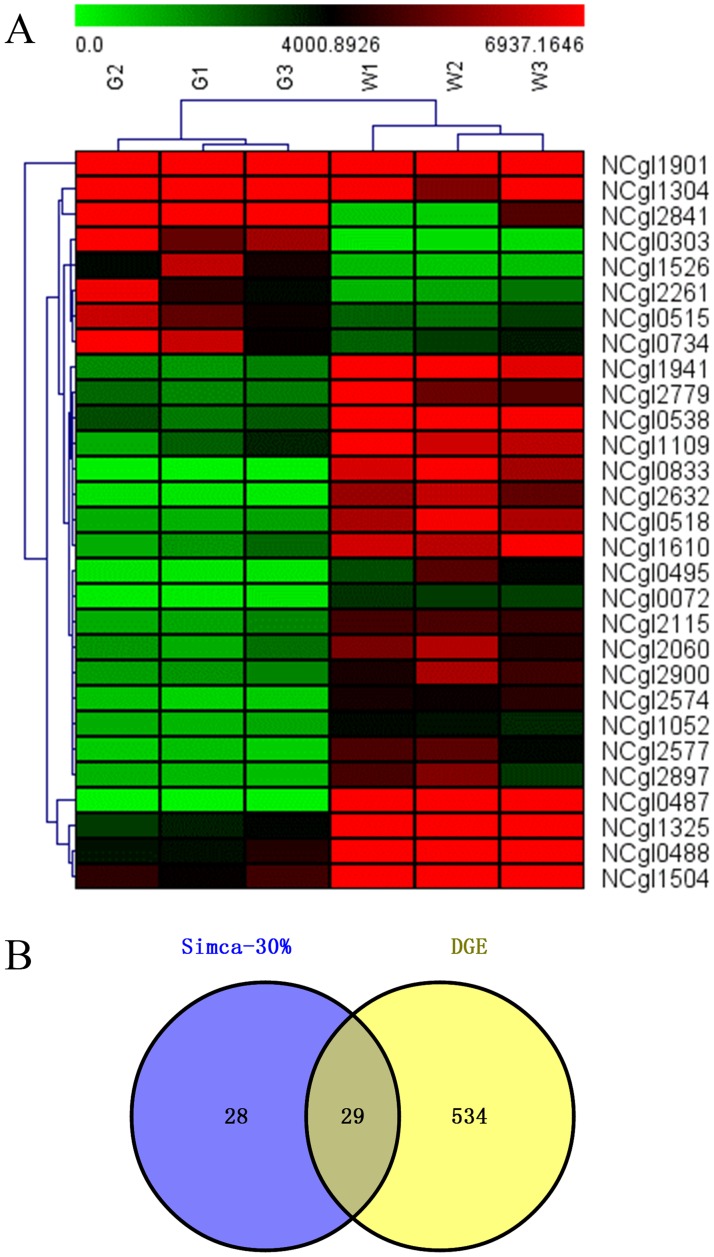
Heat map and Venn analysis of critical genes from MVDA and DEG. A: Heatmap of 29 genes from MVDA, G1, G2 and G3 mean three replicates of *C*. *glutamicum* EGFP; W1, W2 and W3 mean three replicates of *C*. *glutamicum* BZH 001; B: Venn analysis of MVDA and DEG.

DEGs were identified at a false discovery rate (FDR) lower than 0.05 and an absolute value of the log2 ratio≥1 was set as the threshold. However, many critical genes regulated by EGFP expression may be missed through such a screening method. Thus, we applied MVDA to further analyze the RPKM data, and OPLS-DA was performed to identify the critical genes. The S-plot model was used to screen the DEGs of the groups, and the data analysis method of Par scaling was adopted to identify biomarkers. The p1-axis describes the influence of each X-variable on the group separation, and the p(corr)1-axis represents the reliability of each X-variable to accomplish the group separation. Fifty-seven genes were obtained as potential biomarkers from the S-plots, and the venn diagram showed that there were 29 common critical genes from MVDA and DEG analysis of *C*. *glutamicum* EGFP compare with those of *C*. *glutamicum* BZH 001 (Fig 2, S4 Table). There were 10 genes that were hypothetical proteins. Eleven genes encoded ribosomal proteins (NCgl1304, NCgl0538, NCgl1901, NCgl2261, NCgl0518, NCgl0515, NCgl0495, NCgl0487, NCgl0833, NCgl1325, NCgl0488); however, most of them were down-regulated under *C*. *glutamicum* EGFP, indicating that the translation process was suppressed with EGFP expression. There were two genes (NCgl0303, NCgl1526) up-regulated in *C*. *glutamicum* EGFP. NCgl0303 encoding cold shock protein had been discussed above. NCgl1526 encoding glyceraldehyde-3-phosphate dehydrogenase, a house-keeping gene, was up-regulated by 6.61-fold. With the up-regulation of NCgl1526, more ATP and NADH were generated. The other genes are related to the ETC pathway, ABC transporter system, transcriptional regulator and transcription: NCgl2115 encoding cytochrome C oxidase subunit II; NCgl2060 encoding ABC transporter ATPase; NCgl2779 encoding esterase; NCgl1109 encoding helicase; NCgl2897 encoding starvation-inducible DNA-binding protein; NCgl1504 encoding transcriptional regulator. Most of them were related to energy metabolism; however, their relationship with recombinant protein expression should be further studied.

### Analysis of the growth properties of *C*. *glutamicum* BZH 001 and *C*. *glutamicum* EGFP

**Fig 3:**
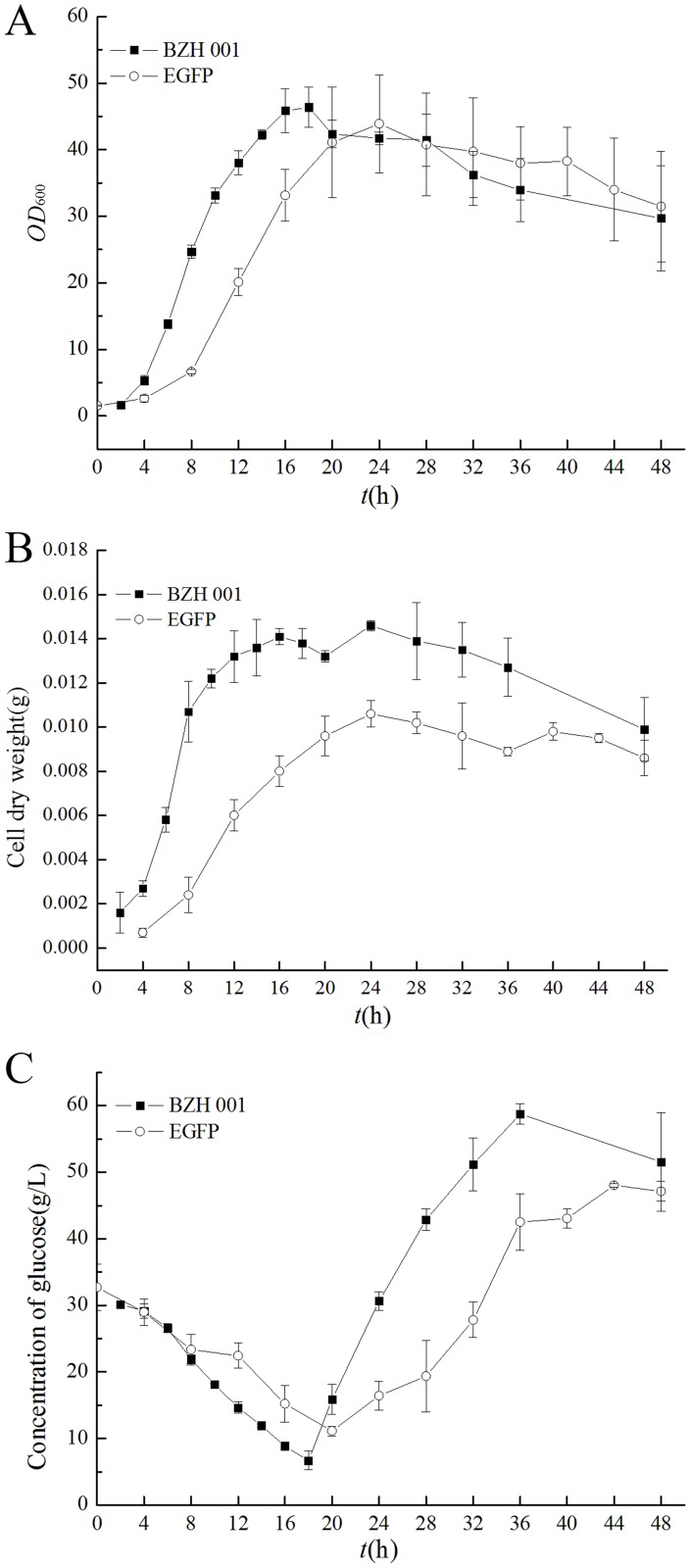
Biomass, CDW and sugar concentration of *C*. *glutamicum* BZH 001 and *C*. *glutamicum* EGFP during 48 h of fermentation.

The expression of heterologous genes is usually a strong burden to the host cells, resulting in a decline in the biomass of bacteria. Fig 3 shows the comparison of *C*. *glutamicum* BZH 001 and *C*. *glutamicum* EGFP concerning the aspects of biomass, cell dry weight (CDW) and sugar consumption (the EGFP expression level was shown in S2 Fig). Both strains grew fast in the initial fermentation stage. The biomass of *C*. *glutamicum* BZH 001 reached its maximum (OD_600nm_ = 46.44 at 18 h and then declined to 29.70 at 48 h), whereas the biomass of *C*. *glutamicum* EGFP reached its maximum at 24 h of 43.92 and fell to 31.47 at 48 h. It was obvious that the heterologous protein expression inhibited cell growth at the early stage because *C*. *glutamicum* EGFP grew poorly compared with *C*. *glutamicum* BZH 001, with a lower max-OD_600_ and longer time to reach the max biomass (Fig 3A). Therefore, it can be reasonably presumed that heterologous protein expression competed with the host-cell growth for the precursors of the engineered strain, resulting in a lower cell growth rate. Fig 3B shows the CDWs of different strains. The CDWs of *C*. *glutamicum* EGFP were lower than those of *C*. *glutamicum* BZH 001 and were kept stable in the middle to late stages. The CDWs of both strains arrived at the maximum at 28 h. We further analyzed the consumption of sugar and found that the sugar consumption rate of *C*. *glutamicum* EGFP was lower before 20 h; however, after 20 h, it was higher than that of *C*. *glutamicum* BZH 001 (Fig 3C). The results indicated that the expression of foreign proteins not only affected the cell growth but also increased glucose consumption to supply more energy for protein synthesis. The NCgl1858 encoding phosphoenolpyruvate-protein kinase, which is a critical gene in the PTS system, was up-regulated by 2.98-fold, and NCgl1305 and NCgl2553 encoding the phosphotransferase system IIC component were up-regulated by 4.54 and 3.86-fold, respectively, both consistently showing the up absorption and transfer of glucose. Increased glucose consumption during the anaerobic condition generally has been considered to produce ATP, and a previous study also reported that increased TCA cycle flux and ATP production were shown in heterologous protein expression in *Pichia pastoris* [27].

### Analysis of the concentrations of amino acids and organic acids of *C*. *glutamicum* BZH 001 and *C*. *glutamicum* EGFP

Many studies have investigated the metabolic pathway for the production of heterologous proteins and/or biomass. The models to express different heterologous proteins were analyzed; these models strongly depended on the amino acid composition of the expressed protein, which has a profound effect on the number and identity of possible pathways for the production of these proteins [27, 28]. Vijayasankaran et al. found that the amino acid composition of heterologous proteins had a profound impact on the predicted elementary flux modes leading to high protein production in *Escherichia coli* [29]. Most of these studies were focused on metabolite drain from the central metabolism for each required amino acid and the energy requirements for the production of each peptide bond. However, we investigated the component of different amino acids and organic acids when EGFP was expressed in *C*. *glutamicum*. Table 1 shows the concentration of amino acids and organic acids of two strains at 48 h. Both the intracellular and extracellular contents of glutamic acid and alanine were higher than those of other amino acids. The extracellular glutamic acid content of both strains was higher than the intracellular production, reaching 2.93 and 3.46 g·L^-1^, respectively. Both the intracellular and extracellular content of glutamic acid of *C*. *glutamicum* EGFP were higher than those of *C*. *glutamicum* BZH 001. Moreover, the extracellular contents of Gly, Val and Met were detectable in *C*. *glutamicum* EGFP, whereas little was detectable in *C*. *glutamicum* BZH 001, which may be caused by the up-regulation of glycolysis and TCA shunting with EGFP expression. The lactic acid and acetic acid contents were greater in *C*. *glutamicum* EGFP than in *C*. *glutamicum* BZH 001, which is generally considered an imbalance between the glucose uptake and up-regulation of glycolysis and the limited activity of the TCA cycle [30]. Moreover, acetate has adverse effects on the synthesis of DNA, RNA, proteins, and recombinant protein could be affected by acetate even at concentrations as low as 0.5 g/L [28, 31]. Six genes involved in glycolysis (NCgl1202 encoding 6-phosphofructokinase, NCgl1526 encoding pyruvate kinase, NCgl2008 encoding glyceraldehyde-3-phosphate dehydrogenase and NCgl2673 encoding fructose-bisphosphate aldolase, NCgl1525 encoding phosphoglycerate kinase, NCgl2008 encoding pyruvate kinase) were up-regulated in *C*. *glutamicum* EGFP. However, the gene NCgl0355 encoding dihydrolipoamide dehydrogenase was down-regulated in *C*. *glutamicum* EGFP, indicating that the TCA pathway was down-regulated, resulting in an anaerobic-like metabolite. To gain sufficient ATP, glycolysis was up-regulated, but the TCA cycle was down-regulated due to the lack of an electron acceptor [32]. The changed contents of amino acids and organic acids may be related to the amino acid composition of EGFP and the demand of ATP in *C*. *glutamicum* EGFP.

**Table 1:** Concentrations of amino acids and organic acids of *C*. *glutamicum* BZH 001 and *C*. *glutamicum* EGFP at 48 h.

Name	Strains	Intracellular content (g·g^-1^)	Extracellular content (g·L^-1^)
Glu	BZH001	0.44±0.012	2.93±0.008
	EGFP	1.26±0.036	3.46±0.011
Ala	BZH001	1.08±0.021	1.70±0.017
	EGFP	0.09±0.007	1.35±0.013
Gly	BZH001	—	—
	EGFP	—	0.31±0.004
Ile	BZH001	—	—
	EGFP	0.08±0.003	—
Val	BZH001	—	—
	EGFP	—	0.76±0.014
Met	BZH001	—	—
	EGFP	—	1.15±0.021
Lac	BZH001	—	0.06±0.003
	EGFP	0.21±0.018	1.38±0.021
Ace	BZH001	—	0.41±0.17
	EGFP	0.32±0.016	1.76±0.012

Abbreviations: Glu: glutamic acid; Ala: Alanine; Gly: Glycine; Ile: Isoleucine; Val: Valine; Met: Methionine; Lac: Lactate; Ace: Acetate; BZH001: *C*. *glutamicum* BZH 001; EGFP: *C*. *glutamicum* EGFP

### Analysis of the enzyme activities of *C*. *glutamicum* BZH 001 and *C*. *glutamicum* EGFP

**Fig 4:**
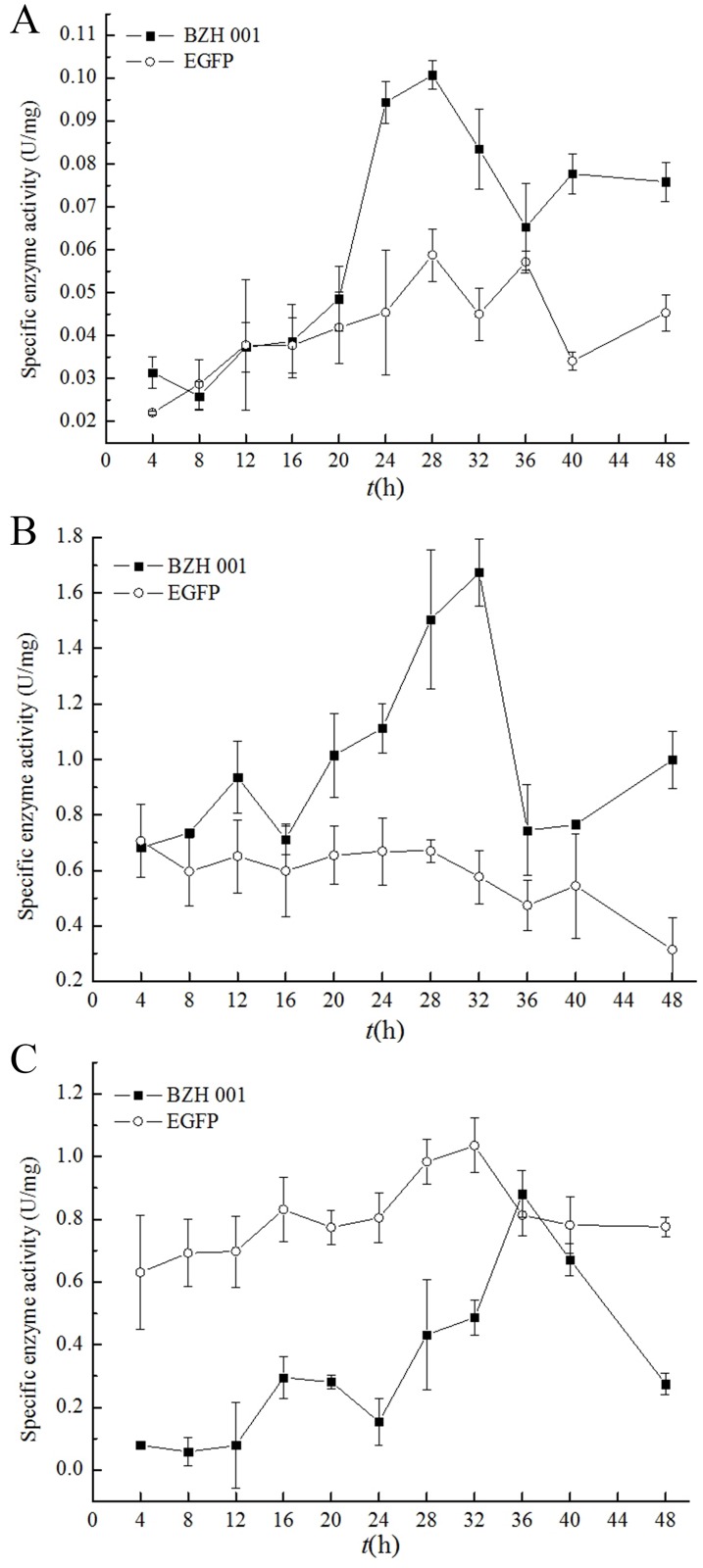
The comparison of specific enzyme activities between *C*. *glutamicum* BZH 001 and *C*. *glutamicum* EGFP. A: PC; B: ICDH; C: GDH.

Enzyme activity is one of the symbols reflecting the intensity of metabolic pathways. The regulation of enzymes directly affects the regulation of metabolic flux of the path, and it is the most basic metabolic regulation. We can indirectly assess the strength of the metabolic pathway by detecting enzyme activities. Enzyme activities were determined for selected carboxylating and decarboxylating reactions of the central metabolism of *C*. *glutamicum* BZH 001 and *C*. *glutamicum* EGFP including the TCA cycle and glutamate synthesis. One of the most important anaplerotic reactions was catalyzed by pyruvate carboxylase. Fig 4A shows a little higher pyruvate carboxylase (PC) activity of *C*. *glutamicum* BZH001 than *C*. *glutamicum* EGFP at 20 h. However, after 20 h, the PC activity of *C*. *glutamicum* BZH001 grew more quickly than that of *C*. *glutamicum* EGFP, reaching 0.1 U·mg^-1^. NCgl0659 encoding PC was 2.17-fold higher than *C*. *glutamicum* EGFP from RNA-seq data. PC catalyzes the pyruvate acid to oxaloacetic acid reaction, and its increased activity supplies more oxaloacetic acid for the TCA cycle at the logarithmic growth phase. However, this process could consume ATP; thus, it was necessary to down-regulate PC activity with EGFP expression. However, the up-regulation of NCgl1523 (encoding phosphoenolpyruvate carboxylase) in *C*. *glutamicum* EGFP may be another way to supply oxaloacetic acid for the TCA cycle and improve the proportion of carbon flux through this anaplerotic reaction, reducing acetate accumulation and improving protein production [28].

Isocitrate dehydrogenase (ICDH) is also an important enzyme in the TCA cycle that catalyzes the generation of α-ketoglutarate from isocitrate and reduces NAD^+^ to NADH. As shown in Fig 4B and 4C, the activities of ICDH in *C*. *glutamicum* EGFP was lower than that in *C*. *glutamicum* BZH 001, indicating that the TCA cycle was weakened in *C*. *glutamicum* EGFP, a finding that was consistent with the result of the analysis of amino acids and organic acids. Glutamate dehydrogenase (GDH) catalyzes the conversion of α-ketoglutarate to Glu. We found that ICDH activity was lower but GDH enzyme activity was higher in *C*. *glutamicum* EGFP than in *C*. *glutamicum* BZH 001, a finding that was consistent with the content of Glu.

### Analysis of the intracellular energy metabolism of *C*. *glutamicum* BZH 001 and *C*. *glutamicum* EGFP

**Fig 5:**
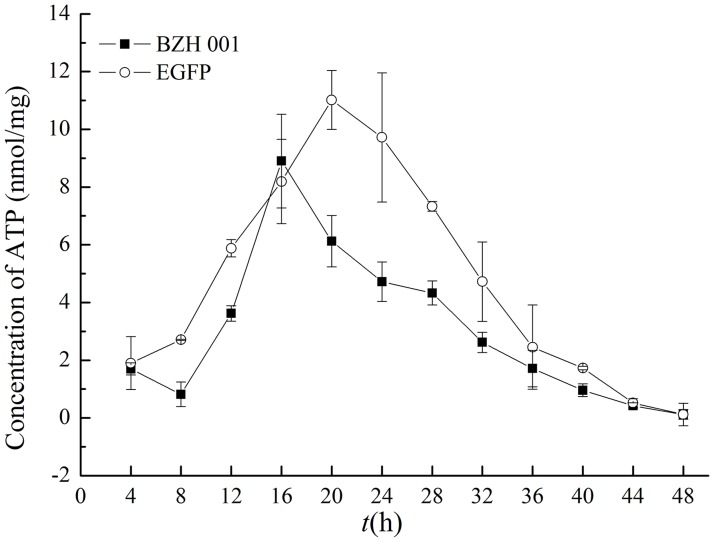
Changes in the ATP content per mg protein of *C*. *glutamicum* BZH 001 and *C*. *glutamicum* EGFP.

As the most important energy source for metabolic pathways, ATP plays a vital role in cell growth and the production of the target products by affecting peptide folding, stress response, transportation, metabolic flux, and signal transduction [33–35]. A prior study had shown that approximately four ATP equivalents were required to form each polypeptide bond of GFP, and the amount of ATP needed from the intermediate metabolism to produce one unit of GFP was 333 [35]. Thus, the requirement of ATP is larger in *C*. *glutamicum* EGFP than in *C*. *glutamicum* BZH 001. In our transcriptome data, atpC, encoding the ATP synthase F0F1 subunit epsilon and NCgl1159, encoding the ATP synthase F0F1 subunit A, were up-regulated. As shown in Fig 5, the ATP content of *C*. *glutamicum* EGFP was higher than that of *C*. *glutamicum* BZH 001 during the entire fermentation process and increased rapidly since 8 h, and the ATP concentration of both strains was reduced quickly after reaching a peak. It could be explained that as a sufficient supply of ATP was essential for the normal metabolism of cells and recombinant protein expression, the metabolic flux was changed to supply sufficient ATP for metabolism and protein synthesis.

## Discussion

**Fig 6:**
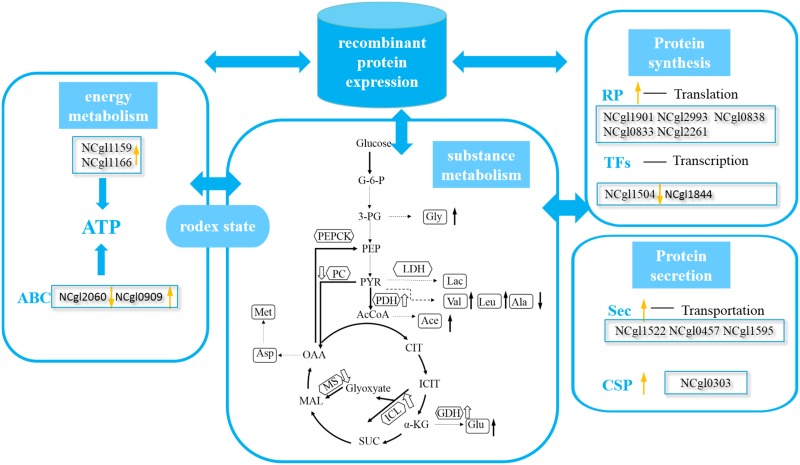
Metabolic shift of *C*. *glutamicum* EGFP under DO 30% concentration. The polygonal boxed enzymes and the hollow arrows indicate significantly up- or down-regulated enzyme activities, respectively. The square boxes represent the metabolites, and the solid arrows indicate the production of significantly up- or down-regulated metabolites, respectively. G-6-P: Glucose-6-Phosphate; 3-PG: 3-Phosphoglycerate; PEP: Phosphoenolpyruvate; PK: Pyruvate kinase; PYR: Pyruvate; LDH: Lactate dehydrogenase; Lac: Lactate; PDH: Pyruvate dehydrogenase; AcCoA: Acetyl-CoA; PC: Pyruvic carboxylase; PEPCK: Phosphoenolpyruvate carboxykinase; OAA: Oxalacetic acid; CIT: Citrate; ICIT: Isocitrate; ICDH: Isocitrate dehydrogenase; α-KG: α-ketoglutarate; GDH: Glutamate dehydrogenase; GLT: Glutamate; Suc: Succinate; MAL: Malate; ICL: Isocitrate lyase; MS: Malate synthase. ABC: ABC transporter system. RP: ribosomal protein. Sec: Sec pathway. CSP: cold shock protein. TFs: transcriptional factors.

We cultivated the wild-type strain (*C*. *glutamicum* BZH001) and engineering strains (*C*. *glutamicum* EGFP) in a bioreactor to study the effect of EGFP expression on metabolism and identify the targets to improve the ability to express foreign proteins in *C*. *glutamicum*. Transcriptome analysis of the response of EGFP expression was carried out for 20 h, and DEG analysis was carried out through KEGG and GO enrichment. MVDA was also used to screen critical genes that play important roles in regulating EGFP expression, and then we detected the content of the intracellular and extracellular metabolites, including organic acids and amino acids, and the activities of the key enzymes in metabolic pathways. Furthermore, we detected the expression of key genes and ATP to study the changes in the material and energy metabolism in the fermentation process. Two effects of EGFP expression were found: 1. metabolism changes in EGFP expression; 2. the effect of EGFP expression on protein synthesis, secretion and expression regulation (Fig 6, S5 Table).

### Metabolism changes for EGFP expression

We found that there was a metabolism change under EGFP expression similar to oxygen limitation (Fig 6). The cause may be that EGFP expression needs more ATP than the wild-type strain to satisfy the consumption of ATP—the metabolism changed to produce more ATP. First, the bacteria of *C*. *glutamicum* EGFP grew poorly compared with *C*. *glutamicum* BZH 001 under the 30% dissolved oxygen condition. The glycolysis pathway was up-regulated with glucose transfer and consumption increase, and more Gly, Lactate and acetate were accumulated under recombinant protein expression [36]; however, the acetate accumulation had adverse effects on EGFP production owning to acetate overflowing is a manifestation of imbalance between glucose uptake and the limited activity of the TCA cycle and respiratory chain, which finally affect the macromolecule synthesis and ATP production [30]. Meanwhile, because of the up-regulation of NCgl2248 encoding isocitrate lyase by 2.95-fold and the ICDH was lower in *C*. *glutamicum* EGFP than in *C*. *glutamicum* BZH 001, the glyoxylate cycle maybe increased. Second, the accumulation of pyruvic acid further leads to the synthesis of more metabolites, including lactic acid, valine and ILe. Third, because more acetate produced leading to carbon overflow of TCA, the increase of phosphoenolpyruvate (PEP) carboxylase (PPC) activity activated the anaplerotic reactions to supply more oxalacetic acid for the TCA cycle. The increase in oxalacetic acid caused more consumption of acetyl-CoA, further leading to decreased acetate accumulation, increased ATP and improved heterologous protein production [37]. In addition, changes in the ATP content are closely related to redox, because of the regulation of each intracellular biological reaction process by intracellular energy levels and the influence of the energy and NADH synergy effect. In the metabolic process of *C*. *glutamicum*, substance metabolism is accompanied by ATP production and redox rebalance.

### Effect of EGFP expression on protein synthesis, secretion and expression regulation

Although the expression of heterologous proteins caused expression changes of many key genes involved in the metabolic and energy pathways of *C*. *glutamicum*, changes in some genes related to protein synthesis and transport should be considered. First, the genes related to ribosomal protein were mostly down-regulated; however, NCgl2261 encoding the 30S ribosomal protein S20 was up-regulated with EGFP expression. The cause may be that the down-regulation of ribosomal protein could save more ATP and substance. Second, the three genes of the sec pathway for proteins transported across the membrane was up-regulated; especially, NCgl1522 encoding secG was up-regulated by 8.2-fold, and a previous study had shown that the overexpression of SecE and SecF could increase the extracellular amylase activity in *C*. *glutamicum* [38]. Additionally, NCgl0303 encoding a cold shock protein belonging to the CspA family was up-regulated by 18.8-fold and may play an important role in the secretion of recombinant protein. A previous study showed that the absence of another protein of the CspA family—CspB—could increase the recombinant Fab secretion from *C*. *glutamicum*. Two genes are related to ABC transporter ATPase—NCgl0909 and NCgl2060. The 31.2-fold up-regulation of NCgl0909 not only affected the activity of the ABC transporter system but also the concentration of ATP; thus, further investigation of this protein is warranted to improve the ATP levels. Third, Transcriptional regulation factor is another factor that impacts recombinant protein expression [39,40]. NCgl1504 is a DeoR family transcriptional regulator; the DeoR-type transcriptional regulator SugR acting as a repressor for genes of the PTS system in *C*. *glutamicum* was investigated [41], showing that DeoR family genes could impact the recombinant protein expression when some genes of the PTS system are repressed. NCgl1844 encodes RNA polymerase sigma factor SigB, whose disruption can increase the secretion of GFP and α-amylase by 3- to 5-fold compared with that in the wild type strain of *C*. *glutamicum* [42]. However, in our transcription data, NCgl1844 showed slight up-regulation. We can down-regulate its expression in a further study for protein expression.

The above results illustrated the influence of heterologous protein expression on metabolism, and many differentially expressed genes related to the metabolic flow changes were found, laying the foundation to identify genomic loci to change the flow of the host cell metabolism. Therefore, research concerning metabolism and the transcriptome regulation of heterologous protein expression in *C*. *glutamicum* has high academic value and broad application prospects.

## Supporting information

S1 Fig The physical map of PXMJ19-EGFP. (PNG)

S2 Fig The EGFP expression level of *C*. *glutamicum* EGFP in the fermentor. (JPG)

S1 Table The specific primer used by real-time PCR. (DOCX)

S2 Table The correlation values and average CV among biological replicates. (DOC)

S3 Table Statistics of alignment analysis. (DOCX)

S4 Table Common critical genes from MVDA and DEG analysis of *C*. *glutamicum* EGFP compared with *C*. *glutamicum* BZH 001. (DOCX)

S5 Table Gene expression level of *C*. *glutamicum* EGFP compared to *C*. *glutamicum* BZH 001. (DOCX)
