# Supplementary figures and images for: Transcriptome analysis of Corynebacterium glutamicum in the process of recombinant protein expression in bioreactors
Source: PLoS One. 2017 Apr 3;12(4):e0174824. doi: 10.1371/journal.pone.0174824 (PMC5378358; doi:10.1371/journal.pone.0174824)

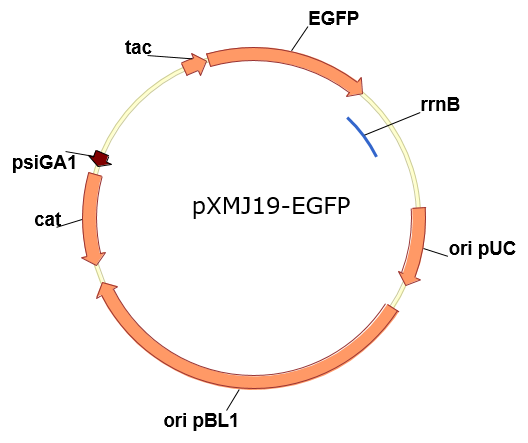

Supplement: S1 Fig — (PNG) [file pone.0174824.s001.png]

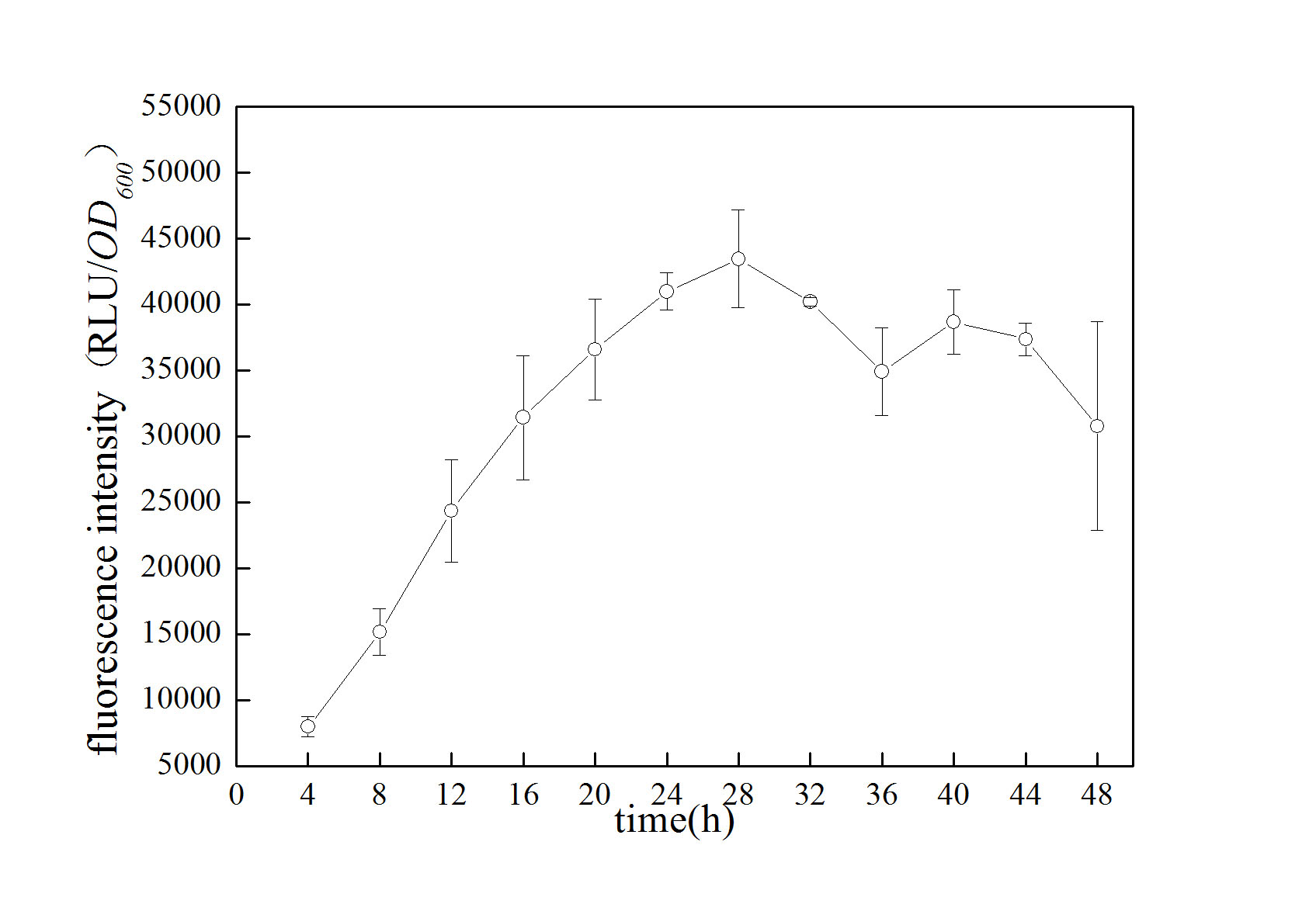

Supplement: S2 Fig — (JPG) [file pone.0174824.s002.jpg]
