# Supplementary material for: Transcriptome analysis of Corynebacterium glutamicum in the process of recombinant protein expression in bioreactors
Source: PLoS One. 2017 Apr 3;12(4):e0174824. doi: 10.1371/journal.pone.0174824 (PMC5378358; doi:10.1371/journal.pone.0174824)
Supplement: S1 Table — (DOCX) [file pone.0174824.s003.docx]

Table S1 The specific primer used by Real-time PCR

| **Gene symbol** | **Sequence(5’→ 3’)** |
| --- | --- |
| *16sRNA* | GTAGGGTGCGAGCGTTGTCC  CGCCATTGGTGTTCCTCCTG |
| NCgl2993 | ATGGCAAAGGGCAAGCG  GACGACGAGCCGCAACAA |
| NCgl0833 | TGCTGGCACTGGTTACACCTA  TACGGACTACTGGATCGTACTTCAT |
| NCgl2261 | ACCGAGATCCGCAAGTTCAA  GGAAGACACCCTTGGTTACTGC |
| NCgl1522 | CTATCGGGCTCCACTGTTGT  GACGCGATCCAGGTTC |
| NCgl0457 | ATGAGGCTAAGCAGGTATCTACAC  TTACGGACTTCTCCCACAAC |
| NCgl1595 | ACAGCACGCAACGGTCATTTC  TTCATTGGCCTTGGACAGCAC |
| NCgl0909 | ATGATGCTGACCACCCACTACCTG  CACGAAACTGATGATCGACTTCTC |
| NCgl0303 | CACAGGGCACTGTTAAGTGGTTC  GTCGGAAGGAGCGATGAAGC |
| NCgl2008 | GTCCCAGACCTGACCCACATC  GCAGAGTAGGAAATAACGCCAC |
| NCgl1858 | GGCTGCGGTCTACTCAAAGG  ATGCGAATGGAACTGGCTTG |
| NCgl2248 | AAGGTGACGGATACATCAACGC  GCCAACCGGACAGGTAGACAG |
| NCgl2632 | AGTTCCGCCGCGAGCAGACTC  TGGTGCGTAGGTGTTGGTGA |
| NCgl1166 | GAACGGTGTCGTGACCATCCAG  GTATCAACCTCATTCGCCCAGAC |
| NCgl1159 | GGAAAGAAGGAAGGTCGTCG  TAACCAGCTACCGCCATCAC |
