# Supplementary material for: Transcriptome analysis of Corynebacterium glutamicum in the process of recombinant protein expression in bioreactors
Source: PLoS One. 2017 Apr 3;12(4):e0174824. doi: 10.1371/journal.pone.0174824 (PMC5378358; doi:10.1371/journal.pone.0174824)
Supplement: S2 Table — (DOC) [file pone.0174824.s004.doc]

Table S2 The correlation values and average CV among biological replicates

| Strian | DO concentration | | Correlation value | | CV(%) |
| --- | --- | --- | --- | --- | --- |
| *C. glutamicum* BZH 001  *C. glutamicum* EGFP | 30% OD | 0.95 | | 1.22 | |
|  | 30% OD | 0.99 | | 0.93 | |
