# Supplementary material for: Transcriptome analysis of Corynebacterium glutamicum in the process of recombinant protein expression in bioreactors
Source: PLoS One. 2017 Apr 3;12(4):e0174824. doi: 10.1371/journal.pone.0174824 (PMC5378358; doi:10.1371/journal.pone.0174824)
Supplement: S3 Table — (DOCX) [file pone.0174824.s005.docx]

Table S3 Statistics of alignment analysis

| Strains | Sample | Total reads number | Total Mapped Reads number and percentage | Unique match reads number and percentage |
| --- | --- | --- | --- | --- |
| *C. glutamicum* BZH001 | 30%DO-1 | 26119252 | 22685543, 86.85% | 22626494, 86.63% |
|  | 30%DO-2 | 27005014 | 23655428, 87.60% | 23583157, 87.33% |
|  | 30%DO-3 | 26616006 | 22921686, 86.12% | 22820106, 85.74% |
| *C. glutamicum* EGFP | 30%DO-1 | 25572552 | 22384649, 87.53% | 22017561, 86.1% |
|  | 30%DO-2 | 22381872 | 19840518, 88.65% | 19644606, 87.77% |
|  | 30%DO-3 | 19321622 | 16518720, 85.49% | 16366182, 84.7% |

30%-OD-1, 2, 3, three biological repeats of samples of strain. Total reads number, total number of sequencing reads; Total Mapped Reads number and percentage, the reads that can aligned to reference sequence and it's percentage; Unique match reads number and percentage, total mapped reads aligned to only one position and its percentage.
