# Supplementary material for: Transcriptome analysis of Corynebacterium glutamicum in the process of recombinant protein expression in bioreactors
Source: PLoS One. 2017 Apr 3;12(4):e0174824. doi: 10.1371/journal.pone.0174824 (PMC5378358; doi:10.1371/journal.pone.0174824)
Supplement: S4 Table — (DOCX) [file pone.0174824.s006.docx]

| Gene ID | Product | Log_2_foldchang |
| --- | --- | --- |
| NCgl1304 | 30S ribosomal protein S1; | 1.020194 |
| NCgl0538 | 30S ribosomal protein S11; | -1.11751 |
| NCgl1901 | 30S ribosomal protein S15; | -1.64593 |
| NCgl2261 | 30S ribosomal protein S20; | 2.106109 |
| NCgl0518 | 30S ribosomal protein S5; | -1.88007 |
| NCgl0515 | 30S ribosomal protein S8; | 1.41533 |
| NCgl0495 | 50S ribosomal protein L29; | -2.90823 |
| NCgl0487 | 50S ribosomal protein L3; | -6.57734 |
| NCgl0833 | 50S ribosomal protein L33; | -4.59583 |
| NCgl1325 | 50S ribosomal protein L35; | -1.69695 |
| NCgl0488 | 50S ribosomal protein L4; | -1.26862 |
| NCgl2841 | hypothetical protein; | 3.060345 |
| NCgl1941 | hypothetical protein; | -1.80811 |
| NCgl2632 | hypothetical protein; | -3.47711 |
| NCgl1610 | hypothetical protein; | -1.2413 |
| NCgl2577 | hypothetical protein; | -2.08293 |
| NCgl2574 | hypothetical protein; | -2.00998 |
| NCgl2900 | hypothetical protein; | -1.16061 |
| NCgl0072 | hypothetical protein; | -2.92537 |
| NCgl0734 | hypothetical protein; | 1.392431 |
| NCgl1052 | hypothetical protein; | -1.16465 |
| NCgl2897 | starvation-inducible DNA-binding protein; | -1.68286 |
| NCgl1504 | transcriptional regulator; | -1.22249 |
| NCgl2060 | ABC transporter ATPase; | -1.24725 |
| NCgl0303 | cold shock protein; | 4.233818 |
| NCgl2115 | cytochrome C oxidase subunit II; | -1.24784 |
| NCgl2779 | esterase; | -1.064 |
| NCgl1526 | glyceraldehyde-3-phosphate dehydrogenase; | 2.704887 |
| NCgl1109 | helicase; | -1.1174 |

Table S4 Common critical genes from MVDA and DEG analysis of *C. glutamicum* EGFP compared with *C. glutamicum* BZH 001
