# Supplementary material for: Transcriptome analysis of Corynebacterium glutamicum in the process of recombinant protein expression in bioreactors
Source: PLoS One. 2017 Apr 3;12(4):e0174824. doi: 10.1371/journal.pone.0174824 (PMC5378358; doi:10.1371/journal.pone.0174824)
Supplement: S5 Table — (DOCX) [file pone.0174824.s007.docx]

Table S5 Gene expression level of *C. glutamicum* EGFP compared to *C. glutamicum* BZH 001

| Gene Function | Gene ID | Name | Product | Log_2_FoldChange (E / WT) | RT-PCR(-ΔΔCt) |
| --- | --- | --- | --- | --- | --- |
| Protein synthesis/ transport | NCgl2993 | *rpmH* | 50S ribosomal protein L34 | 1.74 | 2.63±0.2 |
|  | NCgl0838 | *rpmF* | 50S ribosomal protein L32 | 3.07 | - |
|  | NCgl0833 | *rpmG* | 50S ribosomal protein L33 | -4.60 | -3.0±0.32 |
|  | NCgl2261 | *rpsT* | 30S ribosomal protein S20 | 2.11 | - |
|  | NCgl1522 | *SecG* | preprotein translocase subunit | 3.06 | 4.14±0.15 |
|  | NCgl0457 | *SecE* | preprotein translocase subunit | 2.92 | 3.37±0.03 |
|  | NCgl1595 | *YajC* | preprotein translocase subunit | 1.19 | - |
|  | NCgl0909 | - | ABC transporter ATPase | 4.93 | 6.21±0.31 |
|  | NCgl1937 | - | ABC transporter ATPase | -1.93 | -2.9±0.11 |
| Cold shock protein | NCgl0303 | *-* | cold shock protein | 4.23 | 3.13±0.05 |
| Carbon metabolism | NCgl2008 | *-* | pyruvate kinase | 1.62 | 2.97±0.15 |
|  | NCgl1858 | - | Phosphoenolpyruvate-protein kinase | 1.57 | ± |
|  | NCgl2248 | - | isocitrate lyase | 1.56 | - |
|  | NCgl2632 | - | similar to acetyl-CoA acetyltransferases | -3.48 | -4.0±0.27 |
| Energy metabolism | NCgl1166 | *atpC* | ATP synthase F0F1 subunit epsilon | 1.49 | 1.86±0.09 |
|  | NCgl1159 | *-* | ATP synthase F0F1 subunit A | 1.01 | 1.91±0.02 |
